# Case Report: Benign Uterine Adenomyoma Metastasis in the Right Lung

**DOI:** 10.3389/fsurg.2022.851147

**Published:** 2022-02-22

**Authors:** Xiaowei Zhang, Hongquan Jiang, Bifei Huang, Hangping Wei

**Affiliations:** ^1^Department of Pathology, Affiliated Dongyang Hospital of Wenzhou Medical University, Dongyang, China; ^2^Department of Thoracic Surgery, Affiliated Dongyang Hospital of Wenzhou Medical University, Dongyang, China; ^3^Department of Medical Oncology, Affiliated Dongyang Hospital of Wenzhou Medical University, Dongyang, China

**Keywords:** uterine, adenomyoma, pulmonary, metastasis, benign

## Abstract

**Background:**

Pulmonary metastasis of benign uterine leiomyoma and uterine endometriosis has been reported; however, pulmonary benign metastasizing uterine adenomyoma has not been reported. Herein, we report the first case of pulmonary benign metastasizing uterine adenomyoma. It is very important to differentiate from pulmonary primary synovial sarcoma; histopathology and immunohistochemistry are very helpful, molecular pathology can be used if necessary.

**Case Presentation:**

A female patient was admitted to the hospital because of pulmonary nodules. Lung computed tomography (CT) showed a nodular high density shadow in the upper lobe of the right lung, with a clear boundary and a diameter of approximately 1.2 cm. A contrast CT scan showed obvious enhancement, and no obvious lobulation or burr was found. Video-assisted thoracoscopic resection of the tumor was performed. The upper lobe nodules were completely removed. Postoperative pathological report confirmed the lesion as metastatic benign adenomyoma of the right upper lung.

**Conclusion:**

The lung is the most common organ for malignant tumor metastasis, and a few benign tumors can also develop pulmonary metastasis. Pulmonary benign metastasizing adenomyoma is extremely rare, and the prognosis is very good after surgical resection. When pulmonary CT shows a solid high-density shadow, we should consider the possibility of a metastatic benign tumor.

## Introduction

The lung is the most common region of metastasis. One-fifth of patients with non-pulmonary solid tumors die of lung metastasis, and in a few patients, the lung is the only site of metastasis. Malignant melanoma, osteosarcoma, rhabdomyosarcoma, renal cell carcinoma, germ cell tumor, choriocarcinoma, breast cancer, prostate cancer, and thyroid cancer all have a special tendency for lung metastasis. There are also a few reports of lung metastasis in benign tumors, among which benign leiomyomas are more common in females (1). Herein, we report the first case of pulmonary benign metastasizing uterine adenomyoma confirmed by postoperative histopathology.

## Case Presentation

A 48-years old female was admitted to our hospital on June 20, 2021, because of the discovery of pulmonary nodules in April. When the patient was hospitalized in our hospital 4 months ago, CT showed a clear boundary measuring approximately 1.2 cm in the upper lobe of the right lung. Since the patient had no symptoms of discomfort, regular re-examination was recommended. Therefore, the patient visited our outpatient clinic for re-examination 1 week ago.

## Diagnostic Assessment

### Clinical Features and Imaging

**Figure 1:**
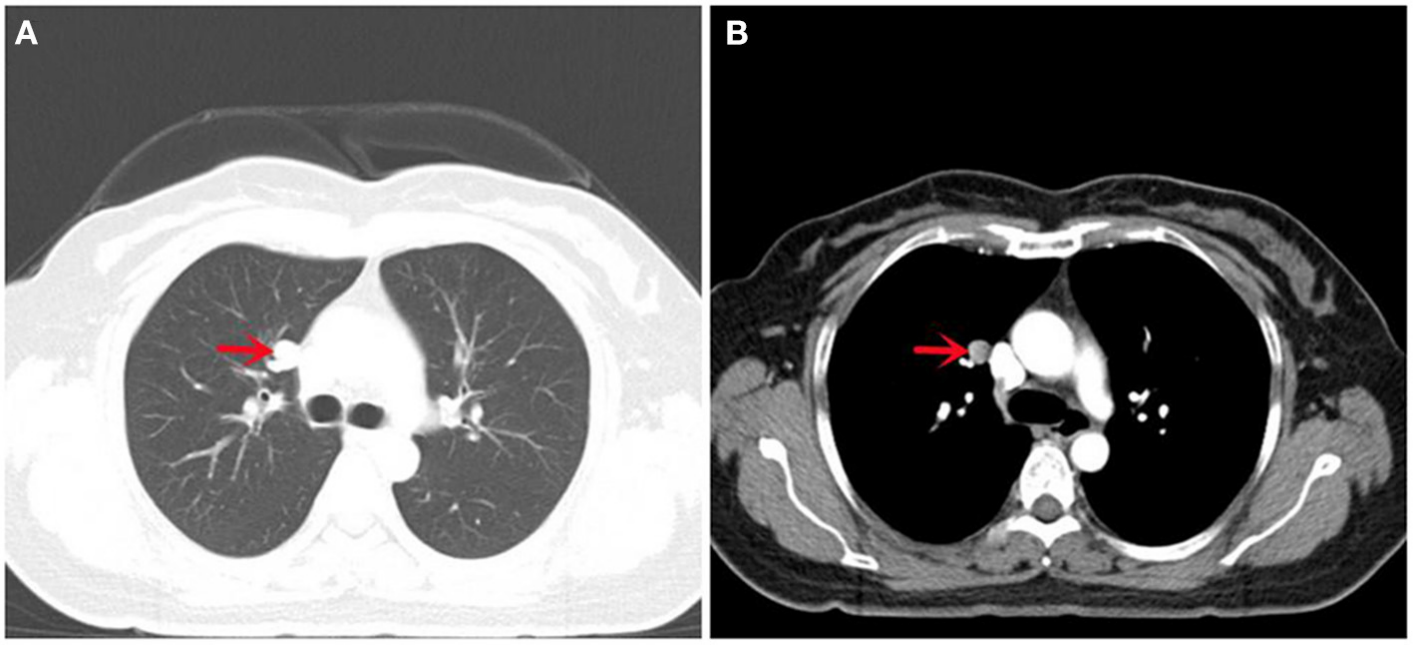
CT images of this case: **(A)** Lung CT showed a round high-density shadow in the upper lobe of the right lung, with clear boundary and smooth edge, without lobulation and burr; **(B)** Enhanced CT of the lung showed significant continuous enhancement.

CT showed a nodular high-density shadow with a clear boundary measuring approximately 1.2 cm in the upper lobe of the right lung, without obvious lobulation and burr (Figure 1A). The enhanced CT scan showed obvious enhancement (Figure 1B). The nodules in the upper lobe of the right lung were similar to those seen initially. The patient had a previous history of laparoscopic resection of leiomyoma in our hospital 5 years ago. During that operation, an extremely large leiomyoma was completely and passively removed. The patient also underwent laparoscopic resection of leiomyoma and adenomyoma in our hospital 4 months ago. The serous layer and myometrium of the uterus at the protrusion of the anterior wall were cut. An adenomyoma-like mass was observed, and the boundary with the myometrium was unclear, a leiomyoma and an adenomyoma were completely and passively removed.

### Treatment

Partial resection of the right upper pulmonary artery under VATS was performed on June 21, 2021. Intraoperative exploration showed that the nodule was located on the mediastinal surface of the upper lobe, with a diameter of 1.4 cm, and there was no change in the pleura. The upper lung was retracted and the anterior mediastinal pleura was opened with an electric coagulation hook, and the upper lobe nodules were completely removed. The patient was followed up for 6 months without recurrence or metastasis.

### Histopathology

**Figure 2:**
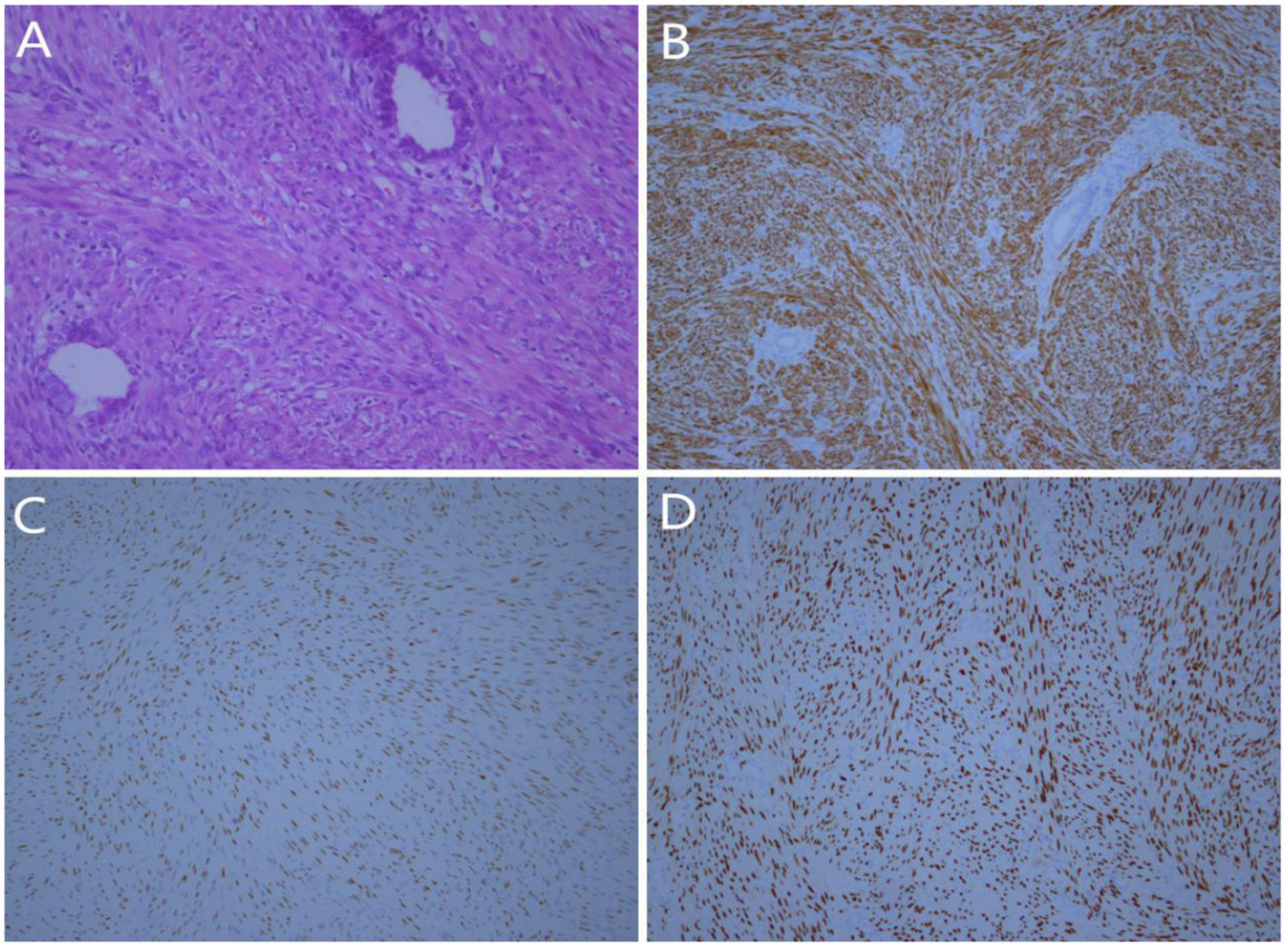
Histologic findings: **(A)** Microscopically, the tumor was composed of proliferative spindled smooth muscle cells and endometrial glands (Hematoxylin and eosin stain, × 200); **(B)** The spindled smooth muscle cells were strongly positive for desmin (IHC, × 100); **(C)** Tumor cells were positive for ER (IHC, × 100); **(D)** The tumor cells were positive for PR (IHC, × 100).

General findings of the specimen were as follows: wedge-shaped resection specimen measuring 5.7 × 4.2 × 3.1 cm, with a peripheral nodule measuring 1.5 × 1.4 × 1.2 cm in the lung lobe, light gray in section, firm, encapsulated, and smooth on the external surface. Microscopically, the nodules were composed of spindle cells, in bundles and in braided arrangement. The tumor cells were round at both ends, and the cytoplasm was eosinophilic and interspersed with a small amount of endometrioid glands (Figure 2A). Immunohistochemistry staining results revealed that the tumor cells expressed desmin (Figure 2B), estrogen receptor (ER) (Figure 2C), progesterone receptor (PR) (Figure 2D), smooth muscle actin (SMA), EMA (expressed in the glands), and Ki-67 was 5%. The specimen revealed negative results for CD10, Cluster of differentiation 34(CD34), S-100, Signal Transducer and Activator of Transcription 6 (STAT6), bcl-2, CD99, HMB45, and Melan-A. The final pathological diagnosis was pulmonary metastasizing uterine adenomyoma.

## Discussion

The lung is the most common organ for distant metastasis of malignant tumors, but there are also a few reports of benign uterine leiomyoma metastasizing to the lung in the literature (1). Benign metastasizing uterine adenomyomas are benign tumors composed of endometrial glands, endometrial stroma, and smooth muscle cells (2). To our knowledge, only 35 cases of extrauterine adenomyomas have been reported (3). The most common sites were the pararectal space, ovary, and broad ligament, other pelvic areas include ligamentum teres, paraovarian, parauterine, and pelvic wall. Extrapelvic adenomas are located in the liver, upper abdomen, inguinal scar, appendix, and mesentery of small intestine (4). Metastasis of uterine adenomyoma to the lung is similar to other uterine tumors. The pathogenesis of metastasis of benign adenomyoma to the lung may be similar to that of benign leiomyoma to the lung. Some people believe that the lesions in the lung are due to the metastasis of uterine lesions through the blood tract. It is also believed that the clone of lung metastatic benign tumors originated from uterine benign tumors (5). In recent years, some researchers believe that during laparoscopic electrosurgical uterine tumor comminution resection, some residual tissue may spread into the abdominal cavity during the removal process, which may increase the risk of extrauterine metastasis (6).

Uterine adenomyomas are usually seen in women of childbearing age, but very few cases occur after menopause (7). The typical clinical manifestations include menstrual hemoptysis or bloody sputum, and most patients with lung uterine benign metastasis adenomyoma will be asymptomatic. Imaging examination is the first choice to detect such lesions, but a definitive diagnosis cannot be made preoperatively (8). The differential diagnosis of adenomyoma should be considered when MRI shows a well-defined mass with a high intensity, bleeding cavity, and concomitant uterine adenomyoma on T1 weighted images (9). This patient underwent myomectomy and adenomyomectomy 4 months before the operation. After partial resection of the right upper lung, the immunohistochemical expression of smooth muscle markers (desmin, SMA) and the expression of ER and PR in endometrial gland and smooth muscle components confirmed the metastasis of uterine adenomyoma to the lung.

This entity should also be distinguished from other tumors with combined mesenchymal and epithelial components (10). Synovial sarcoma of the lung is the main differential tumor, which is characterized by specific chromosome translocation, *t*(X;18)(p11;q11). The immunohistochemical staining (ER/PR positive), myomectomy history, benign leiomyoma most frequently metastasizing to the lung, and presence of endometrioid tissue helps to differentiate. CD34 and STAT6 are positive in intrapulmonary solitary fibrous tumors. Combined with morphology and immunohistochemistry, it is possible to differentiate. Clear cell sugar tumor is a lung mesenchymal tumor, which is composed of clear cells with consistent morphology and can express melanocyte-derived markers (S-100, HMB45, and Melan-A).

Pulmonary metastatic benign adenomyoma of the uterus is a benign tumor. Surgical resection of metastatic lesions is associated with a good prognosis. The treatment of metastatic adenomyoma always follows the treatment principle of uterine adenomyoma, which is usually aimed at reducing the production or induction of endogenous estrogen, progesterone, and adenomyoma differentiation. Gonadotropin-releasing hormone antagonists have been reported to have a beneficial effect on the treatment of adenomyoma (11). It has been reported that elaglix has been successfully used in the treatment of adenomyoma (12). We believe that surgical resection of metastatic lesions is the most effective treatment.

## Conclusion

To our knowledge, this is the first case of a pulmonary benign metastasizing uterine adenomyoma. The lung is a common metastatic site for malignant tumors; however, a few benign tumors can also metastasize to the lung, and clinicians and radiologists should pay special attention to myomectomy and adenomyoma history in female patients. We believe that surgery is the first choice for the treatment of pulmonary metastatic benign adenomyoma.

## Data Availability Statement

The original contributions presented in the study are included in the article/Supplementary Material, further inquiries can be directed to the corresponding author/s.

## Ethics Statement

This study was approved by Affiliated Dongyang Hospital of Wenzhou Medical University Ethics Committee. The patient provided their written informed consent to participate in this study. Written informed consent was obtained from the individual for the publication of any potentially identifiable images or data included in this article.

## Author Contributions

XZ and HW acquired the data. BH analyzed the histological images. XZ, HW, BH, and HJ prepared the manuscript. All authors contributed to the article and approved the submitted version.

## Conflict of Interest

The authors declare that the research was conducted in the absence of any commercial or financial relationships that could be construed as a potential conflict of interest.

## Publisher's Note

All claims expressed in this article are solely those of the authors and do not necessarily represent those of their affiliated organizations, or those of the publisher, the editors and the reviewers. Any product that may be evaluated in this article, or claim that may be made by its manufacturer, is not guaranteed or endorsed by the publisher.

## Abbreviations

CT: Computed tomography
MRI: Magnetic Resonance Imaging
VATS: Video-assisted thoracic surgery
SMA: smooth muscle actin
ER: estrogen receptor
PR: progesterone receptor
CD34: Cluster of differentiation 34
STAT6: Signal Transducer and Activator of Transcription 6
IHC: Immunohistochemistry.
