# Supplementary material for: Case Report: Benign Uterine Adenomyoma Metastasis in the Right Lung
Source: Front Surg. 2022 Feb 22;9:851147. doi: 10.3389/fsurg.2022.851147 (PMC8902040; doi:10.3389/fsurg.2022.851147)
Supplement: Supplementary file 1 [file Data_Sheet_1.doc]

# Consent Form for Case Reports

# 个案报道同意书

Case report: Benign uterine adenomyoma metastasis in the right lung

个案报道：右下肺转移性良性子宫腺肌瘤

You are being asked to consider allowing Dr.Zhang to use information about your disease information to write what is called a case report. This form explains the purpose of this case report. Please read this form carefully and take your time to make your decision and ask any questions that you may have.

你被张医师要求使用你的疾病信息来写一篇病例报告。以下内容是来解释这个病例报告的目的。请仔细阅读此表格。认真做决定,如果你有任何问题，可以随时问。

Dr. Zhang is obligated to protect your privacy and not disclose your personal information (information about you and your health that identifies you as an individual e.g. name, date of birth, medical record number). When the case report is published or presented, your identity will not be disclosed.

张医师有义务保护你的隐私,不透露你的个人信息（这些信息包括你和你的健康信息如名字,出生日期、医疗记录）。但该病例报告发表时,你的身份将不被暴露。

Allowing your information to be used in this case report will not involve any additional costs to you. You will not receive any compensation.

允许使用你的信息在报告中使用，并不会涉及任何额外的成本，你也不会得到任何补偿。

Taking part in this case report is your choice (voluntary). You may choose not to take part or you may change your mind at any time. However, once the case report is written and published, it will not be possible for you to withdraw it. Your decision will not result in any penalty or loss of benefits to which you are entitled including the quality of care you receive.

参加这个案例报告是你的自愿的，你可以选择不参加或者你可能在任何时候改变你的想法。然而,一旦报告已经编辑和出版,你就不可更改了。你的决定也不会引起任何处罚，你也有资格包括接受良好的治疗。

You will be told about any new information relating to this case report that may affect you.

你会被告知任何与你相关新的信息。

Your signature below means that you have read the above information about this Case Report and have had a chance to ask questions to help you understand how your information will be used and that you give permission to allow your information to be used in this case report.

你在下面的签名意味着您已阅读以上信息报告，当然你也有机会提问,可帮助您理解如何信息,同时允许在个案中使用你的信息。

If you have any questions please contact Dr. Zhang.

如果你有任何问题，请联系张医师。

**SUBJECT CONSENT TO PARTICIPATE**

Case report: Benign uterine adenomyoma metastasis in the right lung

个案报道：右下肺转移性良性子宫腺肌瘤

Name of Participant: ___ __________

参加者名字：

By signing this form, I confirm that:

签署该同意书后，我承诺：

- The case report has been fully explained to me and all of my questions have been answered to my satisfaction.

该个案由我解释,所有的问题按照我的意愿来回答。

- I have been informed of the risks and benefits, if any, of allowing my information to be used in this case report

风险及利益已充分告知,如果有的话,我的信息可以被使用在个案中。

- I have read each page of this form

我已经读了这个同意书的每一页。

- I have agreed to participate in this case report

我同意参加这个个案报道。

_______ ____ _______________________ ____ ________

Name of Participant/Substitute Signature Date

Decision-maker (print)
